# Gambling Self-Control Strategies: A Qualitative Analysis

**DOI:** 10.3390/ijerph18020586

**Published:** 2021-01-12

**Authors:** Marie-Claire Flores-Pajot, Sara Atif, Magali Dufour, Natacha Brunelle, Shawn R. Currie, David C. Hodgins, Louise Nadeau, Matthew M. Young

**Affiliations:** 1 Canadian Centre on Substance Use and Addiction, Ottawa, ON K1P 5E7, Canada; mflorespajot@gmail.com (M.-C.F.-P.); satif@ccsa.ca (S.A.); 2 Département de Psychologie, Université du Québec à Montréal, Montréal, QC H3C 3P8, Canada; dufour.magali@uqam.ca; 3 Département de Psychoéducation, Université du Québec à Trois-Rivières, Trois-Rivières, QC G9A 5H7, Canada; natacha.brunelle@uqtr.ca; 4 Department of Psychology, University of Calgary, Calgary, AB T2N 1N4, Canada; scurrie@ucalgary.ca (S.R.C.); dhodgins@ucalgary.ca (D.C.H.); 5 Département de Psychologie, Université de Montréal, Montréal, QC H2V 2S9, Canada; louise.nadeau.2@umontreal.ca; 6 Department of Psychology, Carleton University, Ottawa, ON K1S 5B6, Canada

**Keywords:** gambling guidelines, qualitative, public health messaging, self-control strategies, substance use

## Abstract

There is limited research exploring the perceptions of people who gamble on the self-control strategies used to limit their gambling. This qualitative study examines self-control strategies used to limit money spent gambling, frequency of gambling, and time spent gambling. A total of 56 people who gamble (27 males and 29 females) participated in nine focus groups and five individual interviews in Montreal, Calgary, and Toronto (Canada). Self-control strategies used to limit their gambling expenditure were more common than frequency or time limiting strategies. Strategies to limit expenditure included: restricting access to money; keeping track of money allocated to gambling activities; and avoiding certain types of gambling activities. Various contextual factors were identified to influence those strategies, including social influences; winning or losing; using substances. Findings from this study emphasize the importance of communicating clear gambling limits to people who gamble, as well as the value of developing individual self-control strategies to limit frequency, time and money spent gambling.

## 1. Introduction

Numerous epidemiological studies have shown that problem gambling is affecting up to 5.8% of the general population worldwide, and 2% to 5% of the general population in North America [1]. In 2018, a total of 66.2% of people reported engaging in some type of gambling in Canada, and 0.6% of the population were identified as people with gambling problems [2]. Though legalized gambling is a common activity, a minority of people experience a wide range of harmful consequences as a result [3,4,5].

Gambling may have deleterious consequences or harms. According to Langham et al., a gambling-related harm is defined as “*any initial or exacerbated adverse consequence due to an engagement with gambling that leads to a decrement to the health or wellbeing of any individual, family unit, community or population*.” The authors categorized harms into seven areas of life: financial difficulties, relationship disruptions, emotional or psychological distress, physical health problems, cultural harms, reduced performance at work or study, and criminal activity ([6], p. 4).

People who gamble frequently are at greater risk of experiencing gambling-related harms [7,8]. However, even those who gamble infrequently can also experience adverse consequences as a result of their gambling [9,10,11]. For instance, people without gambling problems have reported impaired control and spending more money and time than originally planned on gambling activities [12].

To minimize gambling-related harms, governments, state agencies and operators have adopted gambling related public health messages (frequently referred to as “responsible gambling”) [13]. Various gambling public health initiatives promote messaging to limit gambling expenditure “not too much” [14,15,16], frequency “not too often”, and time “not too long” [16,17,18,19,20]. Examples of these messages include “bet only what you can afford to lose”; “take breaks on a regular basis”; or “set yourself a time limit”. These types of initiatives have been shown to be moderately effective [15]. However, it is still unclear how these strategies are used by people who gamble, and whether they are perceived as useful and effective.

In order to reduce harm, many people use strategies to minimize negative consequences from gambling. Until now, few studies have examined self-control strategies used by people who gamble to reduce or prevent gambling-related harms. Those that have, have mainly focused on strategies used to limit money spent on gambling [4,21,22,23,24]. For instance, setting monetary limits in advance of gambling is a popular strategy [21,23,25,26]. A mixed-methods study by Rodda et al. found that most EGM (electronic gaming machine) players adopted one or more strategies when planning their gambling activities, such as setting a monetary limit prior to gambling, bringing an exact amount of cash, and not bringing credit cards [24]. Other strategies identified by Bagot el al. to limit or reduce gambling behaviors include behaviour substitution (e.g., exercise instead of gambling), stimulus control (e.g., avoid venues), financial management (e.g., limit access to ready cash), social support (e.g., ask others to support change), and urge management (e.g., postpone gambling) [27].

Another study using an online survey sought to identify self-control strategies among people who gamble regularly experiencing gambling-related harms [4]. From a subsample of 577 participants from Alberta, Canada, nine strategies predicted non-harmful gambling implemented either before or during gambling: (1) stop if not having fun; (2) avoid using money from the household budget; (3) keep a dedicated gambling budget; (4) have a fixed amount that can be spent; (5) engage in other leisure activities; (6) avoid gambling when upset or depressed; (7) not use credit for gambling; (8) avoid gambling to make money, and; (9) not think that strategies can help you win [4]. Notably, most of these strategies involved monetary restrictions.

Within a sample of 10,054 Canadian adults who reported gambling in the past year, the most common strategies reported by survey participants included “setting predetermined monetary limits”, “tracking money spent”, as well as “limiting alcohol consumption while gambling” [21]. Findings from this study highlight, once again, the importance of setting monetary limits to manage gambling behaviors. They also underscore the potential influence of alcohol consumption on self-imposed gambling limits [21]. Although few studies have focused on consumption, they do report that individuals consuming alcohol place higher bets after gambling losses [28] and prolong gambling even when they are losing [29].

A study assessing a variety of gambling related prevention messages such as play summary: “Do you know how much you are spending?” and monetary limit setting: “Have you set your spend limit?” through online focus groups, with four different cohorts of adults who gamble from Manitoba, Canada (*n* = 39), showed that the cohorts differed in their preferences for the type of message wording (e.g., tone) and in the action response (i.e., gambling related prevention tool) that they were prompted to consider [30]. For example, people who gamble on skill-based-games preferred messages that provided information they could incorporate into their own gambling; older adults favored messages on gambling limit setting; and people who gamble frequently and young adults were more responsive to messages about their own play (e.g., through a quiz testing their knowledge of gambling odds) [30]. These results suggest that different types of people who gamble may gravitate toward different self-control strategies.

In short, many studies have identified various self-control strategies used by people who gamble to control their gambling but have mainly focus on monetary expenditure. More strategies suggested in gambling related prevention messages such as time and frequency of gambling need to be investigated.

The research presented is part of a larger project to develop Lower-Risk Gambling Guidelines (LRGGs), led by the Canadian Centre on Substance Use and Addiction (CCSA) [31]. In developing these guidelines, it was important to understand how people who gamble use self-control strategies. The following qualitative study aims to build upon existing gambling literature to explore self-control strategies used by people who gamble, when and why they are used, as well as their perceived effectiveness. An increased understanding of the self-control strategies used by people who gamble as well as the factors influencing them will provide much needed information on how to better adapt existing gambling related prevention and public education messages and develop targeted prevention programs.

## 2. Materials and Methods

Participants were recruited from a sample of 10,054 Canadian adults who participated in a national online survey [32] and reported gambling in the past 12 months. Among this group, 5018 reported using one self-control strategy “at least sometimes”. Respondents who reported playing lotteries only or gambling less than once a month were excluded. Among this group, people who indicated a willingness to participate in further gambling-related research and who resided in Montreal, Calgary or Toronto were contacted and invited to participate. The 332 respondents who met the inclusion criteria were invited via email to participate in this study. Those who responded were redirected to an online participation survey where they selected their language of preference for the interviews, city of residence, choice of retail gift-card and the best way to reach them. Based on participation responses, detailed invitations were sent to 135 potential participants regarding focus groups in their city. Those who were interested but unavailable to participate at that date were invited to an individual interview. The team sought to diversify recruited participants by sex, age, socio-economic status and type of gambling activities, when possible. A total of nine focus groups and five individual interviews were conducted. From these, two focus groups and one individual interview were conducted in French, seven focus groups and four individual interviews were conducted in English.

### 2.1. Procedure

Focus groups and individual interviews took place between 25 April and 20 June 2019. Focus groups were 90 min long and were facilitated by two researchers (MCFP and SA). Interviews were approximately 45 min long and were conducted by one of the two researchers. At the beginning of each focus group and individual interview, the researcher explained the project objectives and asked participants to provide informed consent. Each participant received a list of resources for gambling support and a $50.00 (Canadian) gift certificate for their participation. To ensure participant anonymity, responses are described using pseudonyms. The study protocol was approved by the Research Ethics committee at Université du Québec à Trois-Rivières (CER-19-253-07.18).

### 2.2. Interview Guide

A semi-structured interview guide was used to ensure the main topics of interest were addressed. The guide and research questions were developed and validated in consultation with a group of experts in the field of gambling developing the LRGGs, the Lower-Risk Gambling Guidelines Scientific Working Group (LRGG-SWG). Three main themes were examined: strategies used to control expenditure, frequency, and time spent gambling. In addition, we asked how substance use influenced the use of self-control strategies. A total of 20 open-ended questions were developed, including four warm-up questions (e.g., what are the main types of gambling activities that you do?) to break the ice with participants. The interview guide was designed to encourage discussion. After the warm-up questions, discussions began with questions on the use of self-control strategies (e.g., What do you do to limit how much money you spend gambling?) followed by probing questions regarding the use of substances when gambling (e.g., How are these strategies or tricks impacted when someone is consuming alcohol, cannabis or anything else?). One pilot focus group was conducted in English with a total of four participants, two males and two females, which allowed to test and refine the interview questions and process. This report includes results from the pilot test.

### 2.3. Data Analysis

Data analyses were conducted as focus groups and interviews were completed and recruitment continued until theoretical saturation of the main topics of interest was reached, i.e., until there were no new themes emerging from the discussions [33].

All interviews and focus group discussions were audio recorded and transcribed by a professional transcriptionist. Potentially identifying information (e.g., places) was removed and names of participants were replaced by pseudonyms. A coding grid was developed in collaboration with the co-authors.

Themes were identified and grouped when reading the transcriptions, then merged and prioritized. The data were analyzed using an inductive and deductive thematic analyses approach, using both pre-determined and emerging themes [33]. Pre-determined themes were developed to reflect the objectives of the study, and included strategies used to control expenditure, frequency, and time spent gambling, the use of self-control strategies, and use of substances when gambling. Two researchers (M.-C.F.-P. and S.A.) analyzed the transcriptions using NVivo v.11 for Windows software (QSR International Pty Ltd. Version 11, Ottawa, ON, Canada) One transcription was coded together, and another separately and then both researchers compared and discussed discrepancies. In addition, researchers consulted the study co-authors. Cohen’s Kappa coefficient was used to test interrater reliability. The transcription was tested with the goal of establishing a minimum Cohen’s Kappa of 0.6 interrater reliability, which is considered moderate [34]. An inter-judge Kappa of 0.682 was obtained, thereby rendering the coding grid suitable for analysis. The rest of the transcriptions were divided and coded separately by the researchers. Consultations with study co-authors continued throughout the analysis. The anonymized data collected and analyzed are available to any qualified investigator for audit purposes.

## 3. Results

Participants ranged in age between 23 to 86 years, with an average of 51.9 years old. Participants engaged in a number of different gambling formats including EGMs, lotteries, sports betting and other casino table games, however, the majority reported playing lotteries (Table 1). Table 1 presents the focus groups and interviews by cities.

### 3.1. Self-Control Strategies

In describing self-control strategies participants predominantly discussed ways to limit their gambling expenditure. The strategies participants used to limit money are described below.

#### 3.1.1. Setting Money Limits

Many participants reported setting specific monetary limits when gambling, though specific amounts were rarely disclosed. Furthermore, setting per session limits were common whereas setting a total limit for a week or month was uncommon. When monetary limits were shared, they varied widely among participants or were described as being occasion-dependent. For example, money limits increased when on a trip or when celebrating a special occasion such as a birthday. Furthermore, while some participants set very specific monetary limits, others were more variable in their limit-setting:

*“It isn’t the same amount every time. Sometimes it’s a small amount. I mean, I’ve gone with 60 dollars. And then, there’s other times I’ll go with a couple of hundred dollars.”*

(William, age 52)

Occasionally, participants would set two monetary limits, an initial limit and a second higher limit, just in case they felt “the need” to gamble more or to try to win back losses:

*“Bring so much money with you, but then I think if you’re losing, take a little bit more out, but limit that. So, give it a second chance maybe.”*

(Janet, age 57)

Participants who played lotteries were more likely to set specific monetary limits:

*“I limit myself to about 21 dollars a week. That’s it. Eleven dollars Lotto Max, 10 dollars 6/49.”*

(Maureen, age 62)

*Restricting access to money.* A common strategy most participants reported using, and described as being effective, was restricting access to money. When going to a gambling venue, many participants reported bringing a specific amount of money and leaving their credit and debit cards at home to avoid withdrawing additional money. Restricting access in this way was described as being one of the most effective strategy in limiting the money spent gambling during a session:

*“You leave your credit cards, like you said, credit card, debit cards, leave that in the car or don’t even bring it with you. You bring the amount you want to spend and maybe a little extra that you’re willing to let go and that’s it. So there’s no opportunity to go to the ATM machine or whatever.”*

(Katie, age 54)

*Keeping track of money allocated to gambling activities.* Keeping track of money spent while gambling was another common strategy described by participants. For instance, some used spreadsheets, journals and notebooks to keep track of their wins as well as losses. Participants reported recording their total gambling expenditures helped them stay on track and notice if they overspent. Interestingly, participants stressed how easy it was for them to recall their gambling wins, but not necessarily their losses:

*“You remember the wins. I can tell you how much I won in a night, but I can never tell you what I lose. So, having a spreadsheet, I find, is keeping me accountable, and it’s just a way to feel like I’m not overspending or overdoing anything.”*

(Patricia, age 27)

#### 3.1.2. Setting Time Limits

Participants were asked to describe the strategies used to limit the amount of time spent gambling. It is worth noting that very few of the participants discussed time strategies without being prompted by the interviewers.

*“Usually I set a time limit or whatever. Sometimes we’ll go and play VLTs and then go to a movie or something. So, it’s not just a whole night of gambling. [How much time would you give yourself before the movie?] Maybe an hour. I find it better to limit your time like that, then you’re not tempted to get carried away.”*

(Lisa, age 71)

In fact, the notion of setting time limit was not well understood and had to be explained by the interviewers. Very few reported setting time limits prior to their gambling activities. This may be due to the fact that keeping track of time varies depending on the gambling activity (e.g., lotteries or a poker session). Like expenditure limits, most participants reported using “per occasion” or “per session” as the time frame used to regulate their gambling activities. They were more likely to describe ways in which they limited and kept track of money they spent gambling, as opposed to time:

*“Time wouldn’t work for me really. I don’t use time at all, especially on something if I’m playing sports betting. It’s kind of you’re at the length of the game. So, yeah, it’s hard to really set a time limit. I mean, if I go to play poker, I wouldn’t set a time limit. It’s all based on the amount of money.”*

(Stephen, age 43)

Many felt that setting time limits was not a useful strategy on its own, and should be paired with a monetary limit:

*“I think limiting the money. You know, time…in four hours, you could lose a fortune if you’re on a bad roll. And so, time by itself I don’t know is a very good limiting factor.”*

(Gerald, age 65)

Plan other engagements to limit time spent gambling. Some participants reported intentionally scheduling activities immediately after their gambling sessions to limit the amount of time spent gambling:

*“Like for instance, I’ll be going to the casino tonight. It’s Wednesday night. My wife golfs, I drop her off, and I go to the casino for about two hours, pick her up, and we go for supper.”*

(Ron, age 72)

*Easy to lose track of time while gambling.* Though the majority of participants reported not setting specific time limits to their gambling activities, several shared how easy it was for them to lose track of time while gambling, as they often found themselves “in the zone”:

*“I lose track of time in a casino. It’s something I can’t explain… there’s no windows, you can’t see where it goes from daylight to nighttime. I have walked out of a casino quite surprised that it’s dark out when it was the afternoon when I came in. So, no, I lose track of time in a casino.”*

(Ron, age 72)

#### 3.1.3. Setting Frequency Limits

Participants were asked to describe strategies they used to limit how often they engaged in gambling activities. Similar to time, few discussed these strategies without being prompted by the interviewers. Participants who described adhering to a schedule for their gambling activities were mainly lottery players, as most lottery draws took place on a weekly basis. However, several participants did not report sticking to any particular schedule for their gambling activities and described their frequency pattern as unpredictable:

*“And now that the Lotto Max is on Tuesdays, now it’s like twice a week, so I don’t know. It only recently changed, so only recently have I started spending more than 10 dollars a week. So, now I spend 20, because I always get the Classic Pack.”*

(Brandon, age 47)

*Avoiding online gambling activities.* Some participants reported purposely avoiding online gambling as it is more accessible, and they recognized their vulnerability towards these types of activities:

*“Playing the slot machines online or, I don’t know, card games…I’m not sure exactly what they have…but it just seems so dangerous because there’s nothing to stop you from going and going and going and going. You don’t have to go anywhere. All you need is a credit card and an Internet connection”*

(William, age 52)

*Having obligations or hobbies.* Many participants believed that prioritizing obligations or other hobbies helped them limit how often they engaged in gambling activities:

*“I have a lot of other interests too besides [gambling], like I was saying, I go exercise, all other things, walking, I like to read, I have other activities that keep me busy. I have other things that keeps my interest so I’m not thinking, oh, I have to go gambling.”*

(Katie, age 54)

### 3.2. Factors Influencing Self-Control Strategies

Throughout the interviews, many participants discussed factors they believed influenced one or more of the above-described self-control strategies: the frequency at which they gambled, the time and/or money spent gambling (Table 2). These topics naturally emerged from discussions and are described below.

*Social influences.* Most participants described gambling to be a social activity. Many discussed the influence of others on their gambling behaviors. Some reported their social networks to be valuable in helping them regulate their gambling:

*“Going with somebody else is good because if you tell them ahead of time how much you’re planning on spending, and then you’re going to go over, then they can kind of say to you, “Do you really want to do that?” It hasn’t stopped us, but we do set a certain amount and try and stay at that. And then, I also, my sister and I or whoever will play one machine. So, we have double the money…it lengthens out the time that you can play because you’re both not playing at the same time. So, we just sit and talk and play the machine. So, that kind of helps.”*

(Jessica, age 59)

Others shared that certain people in their social networks led them to spend more time or money gambling:

*“So, it’s pretty strict when I’m by myself. It’s when I’m with other people that’s when I get in trouble. Well, because they may want to stay longer, right? Because they’ll be in a different circumstance. Either they’re winning, or they have more money than I do, and you know, they just want to keep playing.”*

(Jason, age 51)

Some participants mentioned how gambling with others, especially when consuming alcohol, can lead to an increase in time and money spent gambling:

*“You know, whenever you sit around and go somewhere and have drinks with somebody, you sit a little longer, you talk a little more, let’s have another kind of thing. And so then, all of a sudden, well, if you’re having another, let’s play another game, then, kind of thing. So, it just turns into this more social event, which then is going to make you probably spend more money, spend more time.”*

(Catherine, age 57)

*Winning or losing.* Winning or losing seemed to evoke various responses from participants’ regarding their self-control strategies. Several participants had a strict rule of ending a gambling session or “cashing out” as soon as they won any amount in order to walk away a “winner”. Other participants revealed that what deterred them from continuing to gamble was losing money, either big or small amounts:

*“Like if you put a $50 budget and then you put it in the machine and you see in five minutes you run out of 50 bucks it can turn you off and then you say, “Come on, let’s go, let’s go home.”*

(Harry, age 36)

Some participants shared that money won during a gambling session was often used to continue gambling. They reported playing their gambling earnings up until they lost the money they were initially prepared to lose. The wins therefore influenced the initial monetary limits set before a gambling session:

*“Yeah, if I win, I won’t walk away, I’ll play it again until that money that I said I was going to spend”*

(Katie, age 54)

*Feeling lucky and superstitions.* Personal beliefs, particularly regarding luck (e.g., feeling lucky, lucky numbers), appeared to strongly influence some participants’ gambling behaviour. A few shared that they were more likely to gamble on days they “felt lucky” and refrained from gambling if they were experiencing a losing streak. Feeling lucky therefore influenced the limits they set by altering the gambling intentions for the day:

*“Having less luck [make me gamble less frequently] … if I see I’m not winning anything, sometimes I give up.”*

(Gloria, age 33)

Some participants reported consistently playing the same “lucky numbers” to improve their chances of winning the lottery. Others shared some of their superstitious beliefs surrounding the purchase of lottery and scratch tickets. For example, some participants felt the need to buy a ticket that had been touched by a cashier at point of sale, or if it had been left over from a previous sale, just in case it happened to be a winning ticket:

*“I made the mistake of having my numbers like 30 years ago, so I have to play those numbers… The 6/49 used to be one day a week; they went to two days a week. So, I had to play. It’s my birthday number and another magic number… The Lotto 6/49 is the basic that has to be done but then the scratch tickets are just extra, if [the cashier] touched it, then, “Oh, I have to get that one.”*

(Gerald, age 65)

*Ease of access to gambling.* A few participants mentioned how physical proximity to a place of gambling (i.e., casino, corner store, bingo hall, etc.) made it easier for them to gamble more often. For participants unable to drive or access a vehicle, the presence of free public shuttle buses were used. Many expressed concerns regarding online gambling due to its ease of access via smartphone or computer:

*“I used to live part-time in* [Place], *which was close to the casino. And it was easy to get there because I had a ride. In* [new Place], *it takes me almost two hours to get to the casino *[by public transit], *which really makes you think about whether you want to do it or not.”*

(Jessica, age 59)

*Size of potential winnings.* Many participants reported gambling more or going above their regular limits if they deemed the lottery jackpot amount to be higher than usual:

*“I usually buy when I see the jackpot is so large, and it’s attractive enough for me to buy.”*

(Pamela, age 40)

*Special occasions.* Many participants mentioned having different monetary limits for gambling on special occasions. For example, gambling in Las Vegas was considered to be a valid reason for participants to increase their usual gambling limits:

*“I guess it depends where I am. So, if it’s just going to a casino here, it’d be like 100 dollars, but if I was in Vegas, it would probably be like 200 dollars.”*

(Ryan, age 58)

*Receiving work pay.* Several participants reported that receiving their paycheck from work was an incentive for them to gamble more money on that particular day or week. Receiving a paycheck appeared to influence participants’ predetermined monetary limit set for that day:

*“I’ll play when it’s payday. That gives me an incentive to go. You have that extra money, so it goes hand in hand.”*

(Janet, age 57)

*Incentives offered by gambling providers.* A few participants revealed that casinos and gambling websites often gifted them with coupons, free meals or birthday discounts in an attempt to encourage them to either visit their gambling venue, gamble more money, or to stay longer. Incentives offered by gambling providers were therefore reported to increase the frequency of gambling, as well as the time and money spent gambling:

*“Well, you spend 10 dollars, and then when you get to the casino, they give you a 10-dollar coupon. So, it’s basically free. But the trick with that is, you pay, and you get on, and then they give you a voucher to come back, but it’s five hours later. So, they force you to stay there for five hours.”*

(Laura, age 67)

*Mood.* A few participants mentioned that their mood would often influence their gambling:

*“It depends on the day. There are certain days that you might feel happier. So you might go today and you put 20 bucks and another day you might wake up very happy and then you put a hundred or so. Sometimes it really depends on the day and on your humor.”*

(Harry, age 36)

*Witnessing someone experience gambling harms.* Some participants reported that witnessing someone experiencing gambling-related harms, or signs of problem-gambling would often be a deterrent to gambling and would make them gamble less:

*“It’s when that person that is getting addictive is starting to ask you to borrow money to play more, so now you’re getting turned off, you don’t want to gamble anymore, you don’t want to go out with that person anymore”*

(Harry, age 36)

### 3.3. Substance Use and Gambling

During the focus groups and interviews, use of alcohol, cannabis, or other drugs when gambling was not spontaneously discussed. When prompted participants shared their thoughts about the influence of alcohol, cannabis, or other drugs use on gambling in general and not particularly on themselves. Additionally, even though 25% of participants had reported drinking while gambling in the last year, most did not discuss their personal experiences with alcohol; rather, they shared their general perceptions or observations of others they had witnessed gambling.

When asked about the use of substances, most participants mentioned that the consumption of substances such as alcohol or cannabis would impact decision-making such as gambling for longer or spending more money than they intend to, usually leading to negative consequences:

*“If you have a set limit, I think it’s a lot easier to just say, “Oh, I’m going back to the bank machine” when you’ve already had a few drinks. That’s me talking, but … I think the majority of people would be the same.”*

(Donald, age 43)

#### 3.3.1. Alcohol

Most participants believed that alcohol would negatively influence their gambling, as they believed it made them spend more money or more time gambling. Most agreed that consuming alcohol while gambling was a bad idea. Some suggested to set a limit for alcohol consumption when gambling to avoid negative consequences, and a couple even recommended applying the drinking guidelines when gambling:

*“I’m not like a huge drinker to begin with, but yeah, I mean, just probably whatever your limit is for driving is probably more or less a good limit for gambling because it does cloud your judgement, and it can lead to bad decisions. […] So, but yeah, drunk gambling is never a good idea.”*

(Luke, age 39)

Finally, very few participants specifically mentioned how alcohol would affect their self-control strategies, and when they did, they would generally mention that people were less likely to stick to their set limits when consuming alcohol:

*“Everything goes down the drain. All your logical thinking goes away, it kind of becomes more emotional... I let go of my logical thinking and I just continue to play. So alcohol, all drugs, alcohol will definitely change the way people play”*

(Tristan, age 55)

#### 3.3.2. Cannabis

Although interviews took place months after non-medical cannabis was legalized in Canada, participants seemed hesitant in discussing this topic during interviews. Very few participants admitted to having consumed cannabis, either medically or non-medically, even though various had reported in the online survey to have consumed cannabis in the past year. Of the few who responded when asked how cannabis or other substances influenced their self-control strategies, most would avoid using it when gambling:

*“Their judgement clearly is…well, I shouldn’t say clearly…often I see someone who’s…you can tell if they’ve been smoking [cannabis] that their judgement is a little clouded, not everybody obviously, but it can lead to bad decisions that I’ve seen in some people… Just people who can’t focus on what they’re doing… I don’t smoke weed very often, and I wouldn’t do it when I was gambling because I can’t focus on things”*

(Stephen, age 43)

A few brought up the fact that cannabis would discourage them from gambling as often, as it would make them sleepy or hungry. Participants mentioned some side effects of cannabis, including dulling of the mind and carelessness when gambling:

*“I’m a long-time marijuana user. I use THC and CBD for pain because I can’t handle opioids. So, but I don’t think I would gamble when I was stoned, [because] it dulls the mind. And I would imagine if I were going to gamble, I would want to be pretty alert”*

(Katie, age 54)

## 4. Discussion

Several gambling related public health initiatives have promoted messages to gamble “not too much”, “not too often” and “not too long” [14,15,16,17,18,19,20]. This study explored self-control strategies used by people who gamble to limit their gambling.

Focus groups and interviews revealed that people who gamble use a variety of strategies to control their gambling, mainly centered around expenditure, including restricting access to money and keeping track of money allocated to gambling. These findings are consistent with previous research, in which tracking and limiting money spent gambling were common self-control strategies used by people who gamble [4,21,24,35,36,37]. A study by Hing et al. (2019) identified that having a budget dedicated to gambling and setting a fixed monetary amount for gambling were also effective self-control strategies in preventing gambling-related harms [4].

Most participants reported setting specific monetary limits ahead of their gambling sessions, yet many shared that they did not always adhere to these limits when gambling. In fact, many participants reported they brought, or planned to bring, extra money with them for gambling, even if this caused them to exceed their predetermined limits. The presence of additional “backup money” indicates there is a difference between participants’ self-imposed limits and actual money spent gambling. As identified in previous research, behavioral intention does not always lead to actual behavior change [38,39]. This also suggests self-reported monetary limits may be lower than actual amounts people spend or plan to spend in practice. In fact, previous studies have found that exceeding personal set-limits was more likely to occur at higher spending levels [10,21]. This challenges the effectiveness of setting monetary limits as a self-control strategy to limit gambling, as self-imposed limits seemed to fluctuate, and adhering to these limits appeared to be flexible.

Furthermore, discussions revealed that most participants set money limits “per occasion” as opposed to keeping track of their gambling expenses on a consistent basis (e.g., daily, weekly, or monthly). Occasions included celebrations, special events, trips, or outings to the casino, and monetary limits seemed to vary accordingly. To promote clear prevention messaging, future gambling related public health messaging should consider using “per occasion” when promoting monetary limit-setting strategies. Furthermore, reminding people to record expenditure on all gambling activities may help them keep track of money allocated to gambling, and allow them to have a more accurate understanding of the financial impact of gambling. However, from a mental health perspective, caution needs to be taken when asking people to contemplate the consequences of their gambling, as this may trigger significant distress or suicidal ideation, especially in people with gambling problems [40,41,42].

Few participants readily discussed strategies used to limit time and frequency of gambling unless probed by interviewers. Of those discussed, scheduling activities following a gambling session was used to limit time spent gambling (i.e., picking up a family member or booking a restaurant reservation), and prioritizing obligations and hobbies helped limit frequency of gambling. The lack of discussion of these strategies may be due to the confusion reported by several participants when asked to describe strategies used to limit time spent gambling. It is possible that participants may not have been familiar with effective strategies to limit time or were unclear about how to implement such strategies when gambling. In fact, many reported they did not feel the need to keep track of time while engaging in certain gambling activities, such as playing lottery, scratch cards, engaging in a poker game, or placing sports’ bets, nor did they find these strategies particularly pertinent to their own gambling. Therefore, educating people on the strategies of limiting time and frequency of gambling may be helpful, however additional research on limiting time while gambling is needed as this strategy appeared to be particularly difficult to understand according to participants. Moreover, for more effective communication, gambling related public health initiatives focusing on messages to gamble “not too long” and “not too often,” should provide examples of gambling types.

Findings from this study will provide additional insight on how to better adapt existing gambling related public health initiatives. The self-control strategies explored in this study, paired with clear quantitative limits developed for the LRGGs, will refine a broad range of existing messaging that currently advise people to “set a limit and stick to it”. Furthermore, these findings will provide people with evidence-based information to help them make better decisions about their gambling, which is a necessary but insufficient condition to reduce gambling-related harms within the population [31]. As some have noted the limitations of gambling related prevention messaging, additional initiatives should be considered, such as adopting a public health approach to prevent and reduce gambling-related harms and encouraging policy change [43].

Though most participants reported self-control strategies helped them gamble within their limits, they also discussed the challenges of adhering to these strategies. Similarly, Currie et al. found that 45% of online survey respondents failed to “adhere to self-determined quantitative limits for spending, frequency, and time spent gambling” [21]. Participants in our study described several factors that influenced compliance with self-control strategies, which appeared to either increase their predetermined gambling limits, decrease them, or both. For instance, both social influences, as well as winning or losing money, either influenced participants to abstain from gambling, or encouraged them to keep playing. Furthermore, erroneous beliefs about gambling (e.g., feeling “lucky”) also appeared to influence self-control strategy use, which is consistent with previous research [44]. Targeting these factors could be complex, as these may either positively or negatively influence peoples’ self-imposed limits. As these topics naturally emerged from interview and focus group discussions, further research is needed to explore the impact of social and contextual factors, as well as gambling fallacies, on compliance with self-control strategies.

Most participants agreed that substance use, specifically alcohol and cannabis, may impact gambling and adherence to self-control strategies. However, even when prompted by interviewers, participants chose to share observations or hypothetical situations of people using substances while gambling as opposed to discussing any of their own personal experiences. While it is possible that participants did not have any experiences to share, it is also likely that they felt uncomfortable disclosing or detailing any information on their substance use in the context of an individual interview or focus group. Even though several participants had indicated they had previously consumed alcohol while gambling in the recruitment survey (see Table 1), a focus group or interview setting did not appear to favour this discussion as no difference on the depth of the topic was observed in either setting. A cross-sectional survey conducted by Earnshaw, Bergman and Kelly (2019) investigated disclosure comfort among individuals in the United States with resolved alcohol and drug problems. The authors found that participants reported less comfort with disclosure with first-time acquaintances and in public settings, as well as with co-workers and in the media [45]. Therefore, alternative data collection methods, such as anonymous surveys with open ended questions, may be better suited to collect information on the use of substances when engaging in gambling activities. As studies have demonstrated that substance use seems to impact gambling behaviors [21], its role in the utilization of self-control strategies is an important consideration that will need further research.

Finally, “gambling-related harms” also appeared to result in confusion, as participants often required further clarification or examples of such harms. This suggests that public health messages should include clear and comprehensive definitions of what is meant by “gambling” and “gambling-related harms” as well as provide concrete examples to ensure better understanding.

## 5. Limitations

One of the strengths of this qualitative study is the diversity of the study sample. However, despite efforts to include as many perspectives as possible, several limitations must be considered when interpreting the results. A major limitation is that participants were selected from an online survey panel who chose to participate in this study. We only selected individuals who gamble regularly to investigate regulation strategies used by this population, therefore the perspectives of people who gamble irregularly, or less than once a year, are not captured in this study. Efforts were made to acquire different perspectives by recruiting participants with different characteristics such as by sex, age, socio-economic status and different type of gambling activities. Nonetheless it is possible that some of the participants were not well represented in focus groups. Another limitation regarding diversity of perspectives is that the focus groups and interviews were conducted in only three Canadian cities with a limited number of francophones. Future studies including people from different cultures, Indigenous people and communities, and non-urban settings across Canada will ensure a better representation.

Given the sensitive nature of the questions, reporting bias could be a concern if some participants did not fully disclose in a public forum the impact of substance use on their self-control strategies, or whether they struggled to implement self-regulation strategies or stick to their gambling limits due to shame, stigma or social desirability. This was partially mitigated by also conducting individual interviews to maintain anonymity that may be compromised in focus groups. Overall, researchers did not notice significant new information emerging from individual interviews. Yet, as previous research has shown variable effectiveness of gambling related public health messaging across different groups of people who gamble, future work of this nature should consider soliciting the perspectives of people who gamble on skill-based games and young adults [30] to better understand self-control strategies of people who gamble.

## 6. Conclusions

Results from this study provide valuable insights into the self-control strategies used by people who gamble to control money spent gambling, time spent gambling and frequency of gambling, as well as the factors influencing these strategies.

Findings revealed that people who gamble primarily adopt self-control strategies to limit money spent gambling. Furthermore, when people do have strategies to limit gambling expenditure, the limits are “flexible”, and adherence is affected by a wide range of factors. Findings build on previous research by exploring the wide range of factors influencing the efficacy of self-control strategy implementation. Clear messaging is needed to better promote lower-risk gambling. Future research should further investigate factors influencing adherence to self-control strategies as well as the long-term impact of adopting these strategies over time.

## Figures and Tables

**Table 1:** Demographics and gambling profile of participants (N = 56).

Characteristic	Mean (SD) or %
Age (years)	51.9 (14.9)
Sex	
Female	(52%)
Male	(48%)
Total household income ^1^	
Less than 20,000	5%
20,000–39,000	21%
40,000–59,000	11%
60,000–99,000	29%
>100,000	25%
Uncertain or did not to answer	9%
Problem Gambling Severity Index	
Total score	2.9 (5.0)
Non-problem (0)	54%
Low-risk (1–4)	23%
Moderate-risk/Problem (5+)	23%
Consumed alcohol when gambling in the past 12 months ^2^	
Yes	14 (25%)
No	24 (43%)
No response	18 (32%)
Gambling activities (non-exclusive)	
Lotteries	89%
EGMs	20%
Casino table games	13%

^1^ Income in Canadian dollars. ^2^ Respondents who reported drinking alcohol often, sometimes or always were categorized as Yes. Those who reported rarely or never, as No. EGMs = electronic gambling machines.

**Table 2:** Factors influencing the efficacy of self-control strategies.

Factor	Influencing Money Spent Gambling “Not too Much”	Influencing Time Spent Gambling “Not too Long”	Influencing Frequency of Gambling “Not too Often”
Social influences	↑↓	↑↓	↑↓
Winning or losing	↑↓	↑↓	↑↓
Feeling lucky and superstitions	↑		↑
Ease of access to gambling	↑	↑	↑
Size of potential winnings	↑		↑
Special occasions	↑		↑
Receiving work pay	↑		↑
Incentives offered by gambling providers	↑	↑	↑
Mood	↑↓	↑↓	↑↓
Witnessing someone experience gambling harms	↓	↓	↓
Using substances while gambling	↑	↑	↑

↓: Reported to decrease participants’ gambling. ↑↓: Reported to have mixed effects on gambling. ↑: Reported to increase participants’ gambling.

## Data Availability

The data presented in this study are available on request from the corresponding author. The data are not publicly available due to ethical restrictions.
